# Co-expression of PD-L1 and p-AKT is associated with poor prognosis in diffuse large B-cell lymphoma via PD-1/PD-L1 axis activating intracellular AKT/mTOR pathway in tumor cells

**DOI:** 10.18632/oncotarget.9061

**Published:** 2016-04-27

**Authors:** Ling Dong, Huijuan Lv, Wei Li, Zheng Song, Lanfang Li, Shiyong Zhou, Lihua Qiu, Zhengzi Qian, Xianming Liu, Lixia Feng, Bin Meng, Kai Fu, Xi Wang, Qiang Pan-Hammarström, Ping Wang, Xianhuo Wang, Huilai Zhang

**Affiliations:** ^1^ Department of Lymphoma, Sino-US Center for Lymphoma and Leukemia, Tianjin Medical University Cancer Institute and Hospital, National Clinical Research Center of Cancer, Key Laboratory of Cancer Prevention and Therapy, Tianjin, China; ^2^ Department of Pathology, Sino-US Center for Lymphoma and Leukemia, Tianjin Medical University Cancer Institute and Hospital, National Clinical Research Center of Cancer, Key Laboratory of Cancer Prevention and Therapy, Tianjin, China; ^3^ Department of Pathology and Microbiology and Internal Medicine, University of Nebraska Medical Center, Omaha, NE, USA; ^4^ Department of Cellular Biology, School of Basic Medical Sciences, Tianjin Medical University, Tianjin, China; ^5^ Department of Laboratory Medicine, Clinical Immunology, Karolinska Institutet at Karolinska University Hospital Huddinge, Stockholm, Sweden; ^6^ Department of Radiotherapy, Sino-US Center for Lymphoma and Leukemia, Tianjin Medical University Cancer Institute and Hospital, National Clinical Research Center of Cancer, Key Laboratory of Cancer Prevention and Therapy, Tianjin, China

**Keywords:** PD-1, PD-L1, p-AKT, AKT/mTOR signaling, DLBCL

## Abstract

Programmed death-1 (PD-1) /programmed death-ligand 1 (PD-L1) engagement usually leads to diminished antitumor T-cell responses, which mediates the immune escape of tumor cells. However, little is known whether PD-1/PD-L1 could directly activates intracellular oncogenic signaling pathways in tumor cells. The purpose of this study is to investigate whether intracellular AKT/mTOR signaling could be directly activated by PD-1/PD-L1 during the malignant progression in diffuse large B-cell lymphoma (DLBCL). Detection of the expression of PD-L1 and p-AKT by immunohistochemistry (IHC) showed that both proteins were overexpressed in 54% and 48% DLBCL cases, respectively. Spearman test showed that PD-L1 expression was correlated with p-AKT expression (R=0.244, χ^2^=5.962; *P*=0.017) and the expression of PD-L1 and p-AKT were also correlated with clinic-pathological characteristics. In addition, survival analysis showed that DLBCL patients who co-expressed PD-L1 and p-AKT had significantly poorer outcome than patients with single positive or both negative expression (*P*<0.05). In vitro, total PD-L1 and membrane PD-L1 (mPD-L1) proteins were overexpressed in five DLBCL cell lines by western blot and flow cytometry. We observed that AKT/mTOR pathway was activated in DLBCL cells after stimulated with human recombination PD-1/Fc. Taken together, these results suggested that the combination of PD-1/PD-L1 antibodies and AKT/mTOR inhibitor might be a promising and novel therapeutic approach for DLBCL in the future.

## INTRODUCTION

Diffuse large B-cell lymphoma (DLBCL) is the most common type of non-Hodgkin lymphoma (NHL) accounting for approximately 30-40% of newly diagnosed NHL cases [1]. It is a group of highly heterogeneous diseases with variable clinical features and molecular genetic alterations [2]. DLBCL can be classified as two subtypes including germinal center B-cell like (GCB) and activated B-cell like (ABC) DLBCL according to gene expression profiling [3]. Otherwise, DLBCL can be also classified as GCB, ABC and primary mediastinal B-cell lymphoma (PMBL). ABC and PMBL subgroups are grouped as non-GCB DLBCL. Since the addition of the rituximab (R) to cyclophosphamide, doxorubicin, vincristine, and prednisone (CHOP), the prognosis of DLBCL has been greatly improved [4]. However, about 30~40% DLBCL patients still couldn't benefit from R-CHOP and eventually develop relapsed/refractory disease [5]. Hence, further studies are needed to investigate the underlying molecular mechanism of DLBCL and to develop novel therapeutic approaches for this disease.

Programmed death ligand 1 (PD-L1, also known as B7-H1/CD274), a member of the B7 family, is an important ligand of programmed death 1 (PD-1, also known as CD279), which is an immune inhibitory receptor expressed on the surface of T cells, B cells and monocyte upon activation [6]. Recently, PD-L1 was reported to overexpress in different tumors including lymphoma, melanoma, breast cancer, ovarian cancer and bladder cancer [7–11]. Studies also found PD-1/PD-L1 engagement was able to negatively regulate immune response and involved in T cell exhaustion in tumor microenvironment, which promoted tumor progression and metastasis [12–15]. Blockade of PD-1 and PD-L1 has shown to have a therapeutic potential prospect in relapsed/refractory lymphoma and other advanced cancers [16, 17], suggesting that anti-PD-1 or anti–PD-L1 antibodies may be a novel and effective therapeutic approach for lymphoma and other cancers.

Interaction between PD-1 and PD-L1 leads to the suppression of T-cell function and further facilitates immune escape of tumor cells. However, apart from evading host immunity, little is known about the intracellular signal transduction in tumor cell after PD-L1 binding to PD-1. PD-L1 is a type I transmembrane protein with 290 amino acids, consisting of immunoglobulin V-like and C like domains, a hydrophobic transmembrane domain, and a cytoplasmic tail domain [18]. Several studies reported that PD-L2, another ligand of PD-1, directly activated dendritic cell (DC) and functioned as a receptor for signal delivered by cells expressing PD-L2 [19, 20]. PD-L1 and PD-L2 share 34% identity, suggesting they may have similar functions [21]. Meanwhile, it has been reported that PD-L1 appears to be a monomer in solution, but two PD-L1 molecules are present in the asymmetric unit [22, 23], suggesting that it is possible that PD-L1 appears to be a dimer on cell membrane, but monomer in cytoplasm. If a dimer of PD-L1 appears on cell membrane, it is very complex but is indeed possible that a part of PD-L1 could bind to extracellular PD-1, which leads to the suppression of T-cell function, and another PD-L1 could directly activate the intracellular oncogenic signaling pathways. Recently, a very interest study reported that melanoma cell-intrinsic PD-1 could promote tumor growth [24]. This further suggests that a part of the dimer PD-L1 on cell membrane could directly bind to intrinsic PD-1, and then promote the tumor growth. Therefore, we hypothesized that PD-L1 binding to PD-1 could directly activate the intracellular oncogenic signaling pathways in tumor cells. Three oncogenic pathways have been reported in DLBCL, including the constitutively activated NF-kB pathway [25], JAK/signal transducer and activator of transcription (STAT), and AKT/mTOR pathways. These oncogenic pathways usually promote cell proliferation and recede apoptosis [26–28]. Based on these premises, we evaluated whether PD-L1 expression was correlated with phosphorylated AKT (p-AKT) expression and overall survival in DLBCL in this study, and then investigated whether PD-1/PD-L1 binding could directly activate intracellular AKT/mTOR pathway in tumor cells. We found that nearly half DLBCL cases had either PD-L1 or p-AKT overexpression compared to normal lymph nodes, and PD-L1 expression was correlated with p-AKT expression. Co-expression of PD-L1 and p-AKT indicated the worst survival compared to single positive and both negative expression of them. Interestingly, we found that PD-1/PD-L1 binding could directly activate the intracellular AKT/mTOR signaling, not only in T cells, but also in DLBCL tumor cells.

## RESULTS

### Clinical patient characteristics

In this retrospective study, due to lack of tumor tissue or clinical data of some cases, only 100 DLBCL patients between Jan 2008 and Dec 2011 in our hospital were evaluable. Clinical characteristics of the patients were summarized in Table 1. Of the 100 studied patients, there were 60 men and 40 women. The median age was 59 years (ranging from 17 to 85). Forty patients (40%) were diagnosed as GCB-DLBCL and 60 patients (60%) were diagnosed as non-GCB-DLBCL according to the 2008 WHO classification. Thirty-eight patients (38%) were classified as clinical stages I-II and 62 patients (62%) were classified as clinical stages III-IV. Sixty-nine patients (69%) were IPI scores of 0-2 and 31 patients (31%) were IPI scores of 3-5. Thirty-nine patients (39%) were treated with R-CHOP regimen and 61 patients (61%) were treated with CHOP/CHOPE regimen.

**Table 1:** Association of PD-L1 and p-AKT expression with the clinical characteristics of DLBCL

Clinical Parameters	*n*	PD-L1 expression				p-AKT expression			
		Negative (%)	Positive (%)	χ^2^	*P* value	Negative (%)	Positive (%)	χ^2^	*P* value
**Total**	**100**	46(46)	54(54)			52(52)	48(48)		
**Gander**									
male	60	25(41.7)	35(58.3)	1.134	0.312	29(48.3)	31(51.7)	0.808	0.418
female	40	21(52.5)	19(47.5)			23(57.5)	17(48.3)		
**Age (years)**									
<60	52	25(48.1)	27(51.9)	0.188	0.692	33(63.5)	19(36.5)	5.702	0.027*
≥60	48	21(43.8)	27(56.3)			19(39.6)	29(60.4)		
**Pathological pattern**									
GCB	40	24(60.0)	16(40.0)	5.260	0.026*	21(52.5)	19(47.5)	0.007	1.000
non-GCB	60	22(36.7)	38(63.3)			31(51.7)	29(48.3)		
**Clinical stages**									
I~II	38	19(50.0)	19(50.0)	0.395	0.543	21(55.3)	17(44.7)	0.261	0.682
III~IV	62	27(43.5)	35(56.5)			31(50.0)	31(50.0)		
**IPI grades**									
0~2	69	36(52.2)	33(47.8)	3.416	0.084	39(56.5)	30(43.5)	1.823	0.200
3~5	31	10(32.3)	21(67.7)			13(41.9)	18(58.1)		
**Chemotherapy regimens**									
RCHOP	39	19(48.7)	20(51.3)	0.190	0.686	21(53.8)	18(46.2)	0.087	0.839
CHOP/CHOPE	61	27(44.3)	34(55.7)			31(50.8)	30(49.2)		

* *P*<0.05.

### The correlation of PD-L1 and p-AKT expression with clinicopathological features

**Figure 1:**
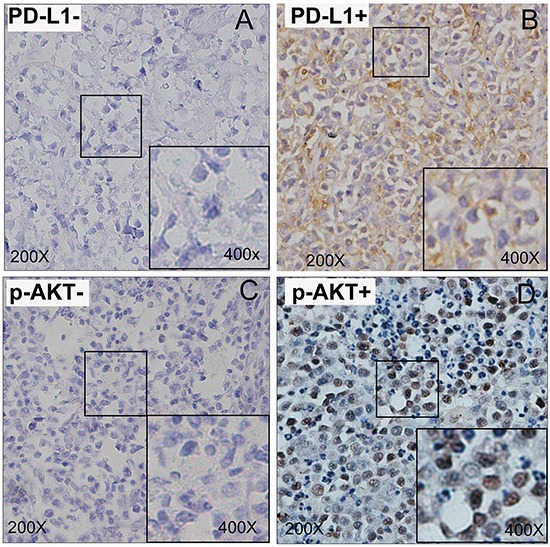
Immunohistochemical staining of DLBCL tumour tissues for PD-L1 and p-AKT expression **A.** Representative patterns of negative PD-L1 expression. **B.** Representative patterns of positive PD-L1 expression. **C.** Representative patterns of negative p-AKT expression. **D.** Representative patterns of positive p-AKT expression.

Positive PD-L1 expression was found in 54 DLBCL cases (54%) by IHC. Representative IHC staining patterns of negative and positive PD-L1 expression were given in Figure 1A–1B. Positive p-AKT expression was observed in 48 DLBCL cases (48%), which was shown in Figure 1D, and representative negative case was also shown in Figure 1C. Co-expression of PD-L1 and p-AKT were observed in thirty-two patients (32%), and thirty patients (30%) showed neither PD-L1 nor p-AKT expression. Twenty-two patients (22%) were positive for PD-L1 expression as well as negative for p-AKT expression. Sixteen patients (16%) were negative for PD-L1 expression as well as positive for p-AKT expression (Table 2).

**Table 2:** The correlation between PD-L1 and p-AKT expression

		PD-L1		Spearman R	χ^2^	*P* value
		negative	positive			
p-AKT	negative	30	22	0.244	5.962	0.017*
	positive	16	32			

* *P*<0.05.

We also found that positive PD-L1 expression was significantly associated with pathological subtype (χ^2^=5.260; *P*=0.026, Table 1) and positive p-AKT expression was significantly associated with older age ≥60 years (χ^2^=5.702; *P*=0.027, Table 1). However, there were no significant difference among other clinical characteristics between positive and negative group for PD-L1 or p-AKT expression (P>0.05, Table 1). Meanwhile, we also found that PD-L1 expression was correlated with p-AKT expression (R=0.244, χ^2^=5.962; *P*=0.017, Table 2).

### The influence of PD-L1 and p-AKT expression on DLBCL patient prognosis

**Figure 2:**
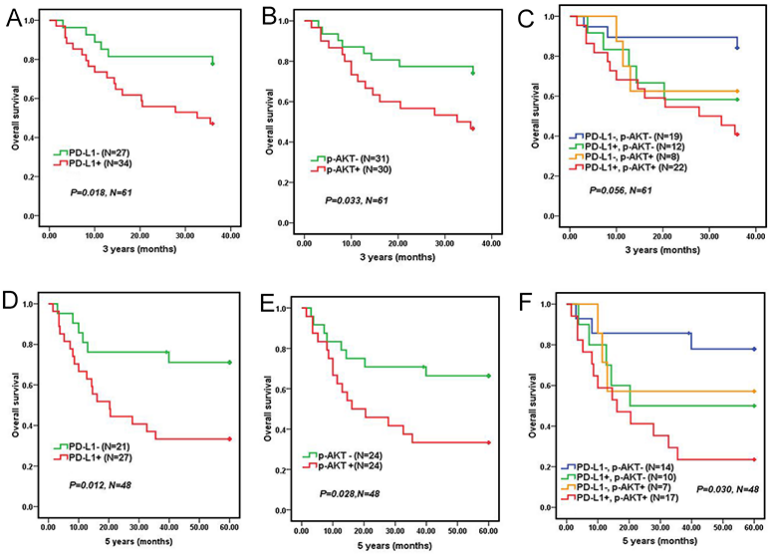
Overall survival of 61 DLBCL patients who adopted CHOP/CHOPE regiment according to PD-L1 and p-AKT expression 3-year overall survival of DLBCL patients with PD-L1 expression **A.** p-AKT expression **B.** and co-expression of PD-L1 and p-AKT expression **C.** 5-year overall survival of DLBCL patients with PD-L1 expression **D.** p-AKT expression **E.** and co-expression of PD-L1 and p-AKT **F.**

Median follow-up duration was 52.4 (ranging from 1.5 to 89.1) months. All of the 100 patients were available for the 3-year overall survival analysis, however only 72 patients could be done for the 5-year overall survival analysis. The results revealed that 3-year and 5-year overall survival rates were 73% and 59.2%, respectively. Clinically, some DLBCL patients were only treated with CHOP/CHOPE. But other DLBCL patients were treated with standard R-CHOP. Considering the influence of different therapy schemes on DLBCL patient prognosis, we separately analyzed 61 patients who adopted CHOP/CHOPE regiment and 39 patients who adopted R-CHOP regiment. Kaplan-Meier survival curves of 61 patients who adopted CHOP/CHOPE regiment showed that DLBCL patients with either positive PD-L1 or p-AKT expression corresponded with a significantly shorter 3 years OS (*P*=0.018, *P*=0.033, Figure 2A and 2B) and 5 years OS (*P*=0.012, *P*=0.028, Figure 2D and 2E) compared to patients with negative PD-L1 or p-AKT expression. Moreover, DLBCL patients with co-expression of PD-L1 and p-AKT had the worst 5 years OS (*P*=0.030, Figure 2F) compared to patients with single positive or both negative expression of PD-L1 and p-AKT, but not 3 years OS (*P*=0.056, Figure 2C). But DLBCL patients with co-expression of PD-L1 and p-AKT had poorer 3 years OS (*P*=0.018) and 5 years OS (*P*=0.007) compared to patients with both negative expression of PD-L1 and p-AKT (Table 3).

**Table 3:** Correlations between PD-L1, p-AKT protein expression with prognosis of DLBCL patients treated with CHOP/CHOPE

Expression	3 years OS				5 years OS			
	Survival rate	Median OS (months)	χ^2^	*P* value	Survival rate	Median OS (months)	χ^2^	*P* value
PD-L1								
negative	77.8%	31.02	5.630	0.018*	71.4%	46.86	6.287	0.012*
positive	47.1%	24.17			33.3%	29.10		
p-AKT								
negative	74.2%	30.11	4.540	0.033*	66.7%	44.50	4.427	0.028*
positive	46.7%	24.20			33.3%	29.25		
Co-expression of PD-L1 and p-AKT								
negative	71.8%	29.43	5.586	0.018*	64.5%	43.31	7.378	0.007*
positive	40.9%	23.25			23.5%	25.15		

* *P*<0.05; OS, Overall survival.

**Figure 3:**
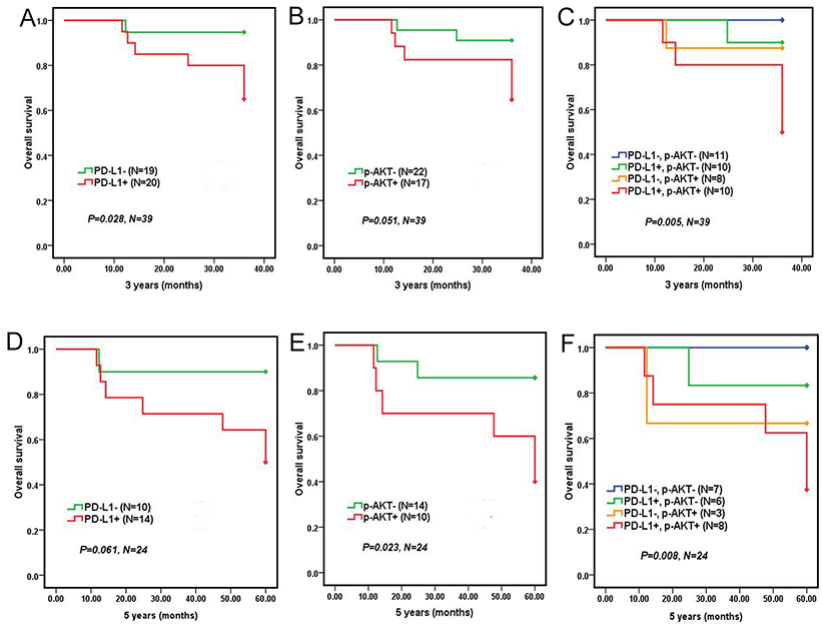
Overall survival of 39 DLBCL patients who adopted R-CHOP regiment according to PD-L1 and p-AKT expression 3-year overall survival of DLBCL patients with PD-L1 expression **A.** p-AKT expression **B.** and co-expression of PD-L1 and p-AKT expression **C.** 5-year overall survival of DLBCL patients with PD-L1 expression **D.** p-AKT expression **E.** and co-expression of PD-L1 and p-AKT **F**.

Kaplan-Meier survival curves of 39 patients who adopted R-CHOP regiment showed that DLBCL patients with positive PD-L1 expression had a significantly shorter 3 years OS (*P*=0.028, Figure 3A), but not 5 years OS (*P*=0.061, Figure 3D), compared to patients with negative PD-L1 expression. However, DLBCL patients with positive p-AKT expression had a significantly shorter 5 years OS (*P*=0.023, Figure 3E), but not 3 years OS (*P*=0. 0.051, Figure 3B), compared to patients with negative p-AKT expression. Moreover, DLBCL patients with co-expression of PD-L1 and p-AKT had the worst 3 years OS (*P*=0.005, Figure 3C) and 5 years OS (*P*=0.008, Figure 3F) compared to patients with single positive or both negative expression of PD-L1 and p-AKT. But DLBCL patients with co-expression of PD-L1 and p-AKT had poorer 3 years OS (*P*=0.002) and 5 years OS (*P*=0.014) compared to patients with both negative expression of PD-L1 and p-AKT (Table 4). Taken together, these results suggested that the co-expression of PD-L1 and p-AKT was associated with worse prognosis in DLBCL patients.

**Table 4:** Correlations between PD-L1, p-AKT protein expression with prognosis of DLBCL patients treated with R-CHOP

Expression	3 years OS				5 years OS			
	Survival rate	Median OS (months)	χ^2^	*P* value	Survival rate	Median OS (months)	χ^2^	*P* value
PD-L1								
negative	94.70%	34.75	4.849	0.028*	87.50%	55.23	3.514	0.061
positive	80.00%	31.97			64.30%	46.50		
p-AKT								
negative	90.90%	34.43	3.804	0.051	84.60%	54.11	5.133	0.023*
positive	82.40%	31.88			55.60%	44.58		
Co-expression of PD-L1 and p-AKT								
negative	93.10%	34.80	9.183	0.002*	87.50%	54.82	6.007	0.014*
positive	50.00%	31.38			37.50%	46.69		

* *P*<0.05; OS, Overall survival.

Subgroup analyses were performed according to gender, age, pathological pattern, clinical stages, IPI scores and chemotherapy regimens, PD-L1 expression, p-AKT expression and co-expression of PD-L1 and p-AKT. The results of univariate and multivariate cox analysis were summarized in Table 5. Univariate Kaplan–Meier estimates demonstrated that age (χ^2^= 5.963; *P*=0.015), pathological pattern (χ^2^=4.823; *P*=0.028), clinical stages (χ^2^=9.470; *P*=0.002), IPI scores (χ^2^=7.645; *P*=0.006), chemotherapy regimens (χ^2^=5.825; *P*=0.016), PD-L1 expression (χ^2^=8.945; *P*=0.003), p-AKT expression (χ^2^=9.246; *P*=0.002), co-expression of PD-L1 and p-AKT (χ^2^=13.992; *P*<0.001) were associated with poor prognosis in DLBCL patients. To determine the prognostic value of PD-L1 and p-AKT expression, multivariate cox regression models for comparison with prognostic factors were applied. We found that clinical stages [HR (95%CI)=3.726 (1.273–10.902), *P*=0.016], chemotherapy regimens [HR (95%CI) =3.564 (1.378–9.217), *P*=0.009], PD-L1 expression [HR(95%CI)=4.740(1.097–20.477), *P*=0.037] and p-AKT expression [HR(95%CI)=6.205 (1.244–30.949), *P*=0.026] were the independent prognostic factors negatively impacting survival.

**Table 5:** Univariate and multivariate analysis of prognostic factors in DLBCL

Clinical Parameters	Univariate analysis		Multivariate analysis	
	χ^2^	*P* value	HR(95%CI)	*P* value
Gander				
Male vs female	0.085	0.77	0.564(0.250-1.271)	0.167
Age(years)				
<60 vs ≥60	5.963	0.015*	1.726(0.661-4.510)	0.265
Pathological pattern				
GCB vs non-GCB	4.823	0.028*	2.392(0.959-5.967)	0.062
Clinical stages				
I~II vs III~IV	9.47	0.002*	3.726(1.273-10.902)	0.016*
IPI grades				
0~2 vs 3~5	7.645	0.006*	0.730(0.269-1.980)	0.536
Chemotherapy regimens				
RCHOP vs CHOP/CHOPE	5.825	0.016*	3.564(1.378-9.217)	0.009*
PD-L1 expression				
Negative vs positive	8.945	0.003*	4.740(1.097-20.477)	0.037*
p-AKT expression				
Negative vs positive	9.246	0.002*	6.205(1.244-30.949)	0.026*
Co-expression of PD-L1 and p-AKT				
Negative vs positive	13.992	<0.001**	0.279(0.041-1.884)	0.190

* *P*<0.05

** *P*<0.001; CI, confidence interval; HR, hazard ratio.

In short, PD-L1 expression was correlated with p-AKT, and the co-expression of PD-L1 and p-AKT would result in a poorer prognosis compared with single positive or both negative expression of PD-L1 and p-AKT in DLBCL. Even PD-L1 and p-AKT were the two independent prognostic factors negatively impacting survival.

### PD-L1 and mPD-L1 expression in DLBCL cell lines

**Figure 4:**
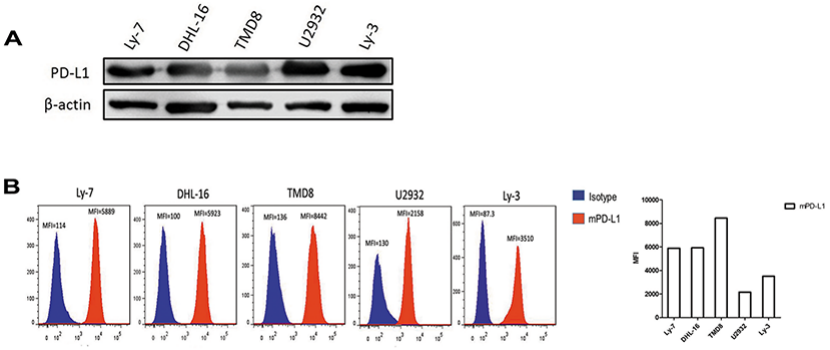
PD-L1 and mPD-L1 expressed widely in DLBCL cell lines **A.** Total PD-L1 protein expression in DLBCL cell lines detected by western blot. **B.** mPD-L1 expression in DLBCL cell lines detected by flow cytometry.

We selected five human DLBCL cell lines to determine the total PD-L1 expression by western blot and mPD-L1 expression by flow cytometry. The results indicated that all of the five DLBCL cell lines expressed PD-L1 (Figure 4A) and mPD-L1 (Figure 4B).

### PD-1/PD-L1 binding directly activates the intracellular AKT/mTOR signaling in DLBCL cells

**Figure 5:**
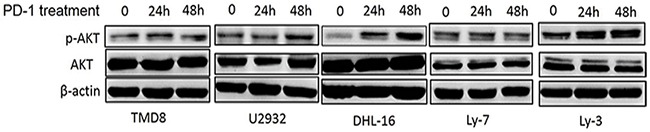
PD-1/PD-L1 binding directly activates the intracellular AKT/mTOR oncogene signaling in DLBCL cells

To further determine whether PD-1/PD-L1 binding could directly activate the intracellular AKT/mTOR signaling in tumor cells, DLBCL cell lines were treated with human recombinant PD1/Fc for 24h and 48h. Total and phosphorylated AKT expressions were evaluated by western blot. Results revealed that p-AKT level was significantly up-regulated in three DLBLC cell lines including DHL-16, U2932 and Ly-3, suggesting that the intracellular AKT/mTOR pathway in DLBCL cells was activated (Figure 5). Taken together, we found that intracellular AKT/mTOR signaling could be directly activated by PD-1/PD-L1 binding in DLBCL cells. It revealed that the combination of PD-1/PD-L1 antibodies and AKT/mTOR inhibitor might be a promising and novel therapeutic approach for DLBCL in the future.

## DISCUSSION

With the advent of rituximab, the OS of DLBCL patients has been significantly improved. However, still 30~40% DLBCL patients inevitably develop into relapsed/refractory disease. So further studies are needed to investigate the underlying mechanism and develop novel therapeutic approaches for DLBCL. Recent studies have highlighted the role of PD-1/PD-L1 pathway in negatively regulating host antitumor response. As the major ligand of PD-1, PD-L1 is widely expressed and correlated with poor prognosis in many human cancers, including melanoma [29], lung cancer [30], glioblastoma [31], breast cancer [7, 32], Wilms’ tumor [33], urothelial cancer [34], pancreatic cancers [35], esophagus adenocarcinoma [36], kidney tumors [37] as well as hematopoietic malignancies [38, 39]. Kiyasu et al have also demonstrated that PD-L1 expression was associated with poor overall survival in DLBCL patients (*P* =0.0323) [39], which was consistent with our results in this study. In our study, we showed that positive PD-L1 expression was associated with poor survival of DLBCL patients for 3-years and 5-years OS (*P*<0.05). Therefore, PD-1 or PD-L1 blockage might be a novel therapeutic approach for DLBCL and other tumors. Several clinical trials carried out in the past two years had revealed that both anti-PD-1 antibodies (such as Nivolumab, Pidilizumab, and Pembrolizumab) and anti PD-L1 antibodies (such as MPDL3280A) had shown significant antitumor effects in patients with metastatic melanoma [40], Hodgkin's lymphoma [16], bladder cancer [41], and lung cancer [42].

DLBCL tumors have several important oncogenic signaling pathways which make DLBCL to be a heterogeneous disease and impact overall survival of DLBCL patients. The AKT/mTOR signaling pathway has been demonstrated to be constitutively activated in DLBCL [28], and AKT plays a central role in signaling transduction. Activated AKT participates in multiple downstream pathways to promote the malignant phenotype of cancer cells through mediating various pro-survival signals [43–46]. As an important downstream target of AKT, mTOR plays a key role in cell cycle progression and metabolism [47]. Xu et al found that activation of AKT/mTOR pathway was related to poor outcome in DLBCL patients treated with CHOP but not R-CHOP [48]. In our study, we found that activation of AKT/mTOR pathway was related to poor survival in DLBCL patients treated with CHOP/CHOPE for 3-years and 5-years OS (*P*=0.033, *P*=0.028), and R-CHOP for 5-years OS (*P*=0.023), but not R-CHOP for 3-years OS (*P*=0.051).

In our study, spearman test showed that PD-L1 expression was correlated with p-AKT expression in DLBCL(R=0.244, χ^2^=5.962; *P*=0.017). In addition, we found that the DLBCL patients who co-expressed PD-L1 and p-AKT had the worst clinical outcome compared with the patients who were single positive or both negative expression of PD-L1 and p-AKT for 3-years and 5-years OS (*P*<0.05). So we hypothesized that PD-1/PD-L1 binding might directly activate the intracellular AKT/mTOR oncogenic signaling in tumor cells to promote DLBCL progression. To confirm this hypothesis, we selected five DLBCL cell lines, which overexpressed PD-L1 protein and mPD-L1, to be stimulated with human recombinant PD-1/Fc protein for 24h and 48h. Interestingly, we found that level of p-AKT protein was significantly up-regulated with a time-dependent manner, suggesting that PD-1/PD-L1 binding could directly activate the intracellular AKT/mTOR oncogenic signaling in DLBCL tumor cells. Otherwise, Parsa et al found that expression of the gene encoding PD-L1 increased after loss of *PTEN* gene and activation of PI3K pathway in human glioma [49]. Lastwika et al found that activation of AKT/mTOR oncogenic pathway promoted immune escape by driving the expression of PD-L1 in NSCLC [50]. Taken together, it was possible that there was a positive feedback loop between PD-1/PD-L1 axis and AKT/mTOR oncogenic signaling.

Our results indicated that the combination of PD-1/PD-L1 antibodies and AKT/mTOR inhibitors might be a promising and novel therapeutic approach for DLBCL in the future. In addition, multivariate analysis in this study showed that expression of PD-L1 or p-AKT was the dependent prognostic factor for DLBCL patients. We also found that PD-L1 expression was related to the pathological subtype, but p-AKT expression was correlated with ages. The reasons for this observed distinction between them were unclear. The numbers of patients included in our study was relatively small, and so these results required further validation in a large cohort. But it showed a consistent trend that DLBCL patients with co-expression of p-AKT and PD-L1 had worse prognosis compared to patients with single positive or both negative expression of PD-L1 and p-AKT, who were treated with either R-CHOP or CHOP/CHOPE. These results suggested that co-expression of PD-L1 and p-AKT was still noteworthy in the rituximab era, and rituximab could not overcome poor prognosis of co-expression of PD-L1 and p-AKT in DLBCL.

In summary, DLBCL patients overexpressed PD-L1 and p-AKT, and co-expression of them showed a significantly worse survival compared to single positive or both negative expression of them. PD-1/PD-L1 binding might activate the intracellular AKT/mTOR oncogenic signaling pathway in tumor cells to promote DLBCL aggressiveness. Thus, a more effective treatment approaches should be developed for this subset of DLBCL patients, and the combination of targeting AKT/mTOR and PD-1/PD-L1 pathway blockade might be a promising therapeutic strategy.

## MATERIALS AND METHODS

### Patients and samples

A total of 100 DLBCL cases with formalin-fixed paraffin-embedded (FFPE) tissues in the Tianjin Medical University Cancer Institute and Hospital (TMUCTH, Tianjin, China) from Jan 2008 and Dec 2011 were studied. Archived FFPE tumor tissues were obtained from our Department of Pathology and these cases were reclassified according to the 2008 WHO classification and Hans algorithm by experienced hematopathologists. In addition, 10 specimens of normal lymph gland tissue obtained from patients with reactive hyperplasia of lymph node were used as normal controls. All clinical information was obtained by reviewing the patients’ medical charts. The study and all protocols below were approved by the Ethics Committee of TMUCTH.

### Immunohistochemistry

IHC staining for PD-L1 and p-AKT proteins were performed using the streptavidin–peroxidase method (SP method). Briefly, the paraffin-fixed slides were dewaxed in xylene and rehydrated through graded alcohols. Antigen retrieval was respectively carried out using EDTA buffer (pH 8.0) for anti-PD-L1 and citric acid buffer (pH 6.0) for anti-phospho-AKT (Ser473) in 120°C for 2 minutes and 30 seconds. Endogenous peroxidase activity was blocked using 0.3% hydrogen peroxide for 10 minutes at room temperature in dark place. Nonspecific binding of the primary antibody was blocked by incubating the slides with 10% normal horse serum for 30 minutes at 37°C. Then they were incubated with the primary antibodies including rabbit anti-PD-L1 polyclonal antibody (1:200, ab153991, Abcam, Cambridge, UK) and rabbit anti-phospho-AKT (Ser473) polyclonal antibody (1:100, AF0908, Affinity Biosciences, USA) at 4°C overnight. And then they were incubated with secondary anti-rabbit IgG/HRP at 37°C for 30 minutes. Subsequently, for visualisation of the antigen, the sections were performed with the chromagen 3, 3′-diaminobenzidine. The slides were counterstained with hematoxylin and mounted under coverslips.

### Evaluation of IHC for PD-L1 and p-AKT proteins

Percentages of PD-L1 positive tumor cells and staining intensity were evaluated for each slide. Staining for PD-L1 was considered high expression, if ≥5% of the tumor cell population showed 2+ or 3+ membrane staining. In addition, if ≥20% of the total tissue cellularity showed 2+ or 3+ membrane or cytoplasmic staining in malignant and/or nonmalignant cells, it was considered to have a microenvironment positive for PD-L1 [51]. p-AKT expression was semiquantitatively assessed based on the staining intensity and the proportion of the stained tumor nuclei cells as follows: staining proportion was also classified as 0–3 (0 = 0–5 %, positive cells, 1 = 5–10 % positive cells, 2 = 10–50 %, 3 = ≥50 %) and the intensity of p-AKT staining was scored as 0-3 grades (0 = negative, 1 = weak, 2 = moderate, and 3 = strong). Only when the score reached 3–9, it represented positive. This scoring system was similar to previous studies [52, 53]. Two independent observers evaluated the expression levels of PD-L1 and p-AKT protein following the evaluation system and both observers reexamined the immunostained slides to determine a consensus score.

### Cell culture

The DLBCL cell lines: TMD8, U2932, SUDHL-16 were cultured in RPMI-1640 medium (Life Technologies, California, USA) containing 10% fetal bovine serum (Hyclone, UT, USA). OCI-LY3 (LY3) and OCI-LY7 (LY7) were cultured in IMDM medium (Life Technologies, California, USA) supplemented with 15% fetal bovine serum (Hyclone, UT, USA). All medium were supplemented with 1% penicillin-streptomycin and all cells were incubated at 37°C in 5% CO2.

### Flow cytometric analysis

For flow cytometry, DLBCL cell lines were harvested, washed, and then labeled with fluorochrome-conjugated PE anti-Human CD274 (PD-L1, B7-H1) (clone 29E.2A3, BioLegend, USA) for 30 minutes at 4°C in dark place. Then the mPD-L1in all samples was measured by flow cytometry (LSDFortessa, BD Bioscience). Results were analyzed with FlowJo software (version 7.6).

### Stimulation of DLBCL-cell lines with human recombinant PD-1/FC

All five cell lines were cultured in complete medium containing 100ug/ml human recombinant PD1/Fc (10377-H03H, Sino Biological Inc., Beijing, China) for 24h and 48h. And then total proteins were isolated and run using western Blot to detect the total AKT and p-AKT expression.

### Western blot analysis

Cells were harvested and suspended in RIPA lysis buffer containing 1 mM phenylmethylsulfonyl fluoride (PMSF). 20-50μg total protein (depending on different proteins) were separated by sodiumdodecyl sulfate–polyacrylamide (SDS–PAGE) gel electrophoresis and then transferred to polyvinylidene difluoride membranes (PVDF, Roche, UK), which were then blocked in 5% non-fat milk in Tris-buffer saline-Tween (TBST) for 1 h. The immunoblotting was performed by incubation with the primary antibodies including PD-L1 (Abcam, Cambridge, UK), p-AKT and total-AKT (Affinity Biosciences, USA), and β-actin (Cell Signaling Technology, Boston, USA), which was used as an endogenous protein for normalization at 4°C overnight. Blots were then washed and incubated with a 1:3000 dilution of Goat anti-Rabbit IgG H&L (HRP)-conjugated secondary (Millipore, Billerica, MA, USA). Signals were detected by enhanced chemiluminescence Plus reagents (Amersham Pharmacia, Piscataway, NJ) and formed images by Chemiluminescence Imaging System. Signal quantification was obtained using Quantity One software (Bio-Rad Laboratories, USA) and normalized to β-actin.

### Statistical analysis

Clinical, pathological, and chromosomal charac- teristics of the patients were compared using chi-square tests or Fisher's exact tests. The Kaplan-Meier method was used to estimate the over survival (OS) distributions, and the log-rank test was performed to compare the survival difference. Univariate analysis were performed with log-rank test. Multivariate-adjusted Cox regression models were used to evaluate independent prognostic factors. Difference was considered significant when the P value was <0.05 and all reported P values were two sided. These statistical analyses were performed with SPSS 17.0 statistical software (Chicago, IL, USA).
